# Moderate-risk variants in hereditary breast and ovarian cancer: seven-year experience from a specialized center

**DOI:** 10.1007/s10689-026-00609-5

**Published:** 2026-10-15

**Authors:** Annika Krückel, Matthias W. Beckmann, Lena Brückner, Peter A. Fasching, Paul Gass, Julia Gocke, Carolin C. Hack, Felix Heindl, Manuel Hörner, Emelie Kunze, Carolin Müller-Wirtz, Niklas Amann

**Affiliations:** 1 Department of Gynecology and Obstetrics, Universitätsklinikum Erlangen Comprehensive Cancer Center Erlangen-EMN (CCC ER-EMN), Erlangen, Germany; Friedrich-Alexander-Universität Erlangen-Nürnberg (FAU), Erlangen, Germany; 2 Bavarian Cancer Research Center (BZKF), Erlangen, Germany; 3 Klinikum Chemnitz gGmbH, Medizincampus Chemnitz der Technischen Universität Dresden, Chemnitz, Germany

**Keywords:** Breast cancer, Ovarian cancer, Genetic testing, HBOC, IBCS

## Abstract

Moderate-risk gene variants represent an increasingly recognized component of hereditary breast and ovarian cancer (HBOC) predisposition, yet their clinical interpretation and management remain less standardized than those of high-penetrance genes. This study provides a seven-year real-world evaluation of genetic variants in *ATM*,* BARD1*,* CHEK2*,* RAD51C*, and *RAD51D* in individuals undergoing germline testing at a specialized HBOC center, with a particular focus on variant distribution and associated clinical management. We retrospectively analyzed germline testing data from 3416 individuals evaluated at the HBOC center Erlangen between 2018 and 2024. Testing indications included predictive as well as diagnostic and therapeutic testing in the context of breast and gynecologic neoplasms. Variant classifications followed ACMG/IARC (American College of Medical Genetics and Genomics/ International Agency for Research on Cancer) criteria. Clinical recommendations, including participation in the intensified breast cancer surveillance (IBCS) program and risk-reducing surgery, were extracted from structured counseling documentation. (Likely) Pathogenic variants in moderate-risk genes were identified across all clinical subgroups. *CHEK2* represented the most frequently altered moderate-risk gene, followed by *ATM*. In contrast, (likely) pathogenic variants in *BARD1*, *RAD51C*, and *RAD51D* were observed solely among individuals undergoing predictive testing or presenting with breast malignancies. A small subset of individuals carried more than one (likely) pathogenic variant in HBOC susceptibility genes. Most women with moderate-risk variants were enrolled in the IBCS program, although in justified cases participation was withheld based on patient-specific risk factors. An isolated moderate-risk variant generally did not warrant a recommendation for risk-reducing breast surgery. Risk-reducing salpingo-oophorectomy (RRSO) was primarily discussed with individuals harboring *RAD51C* or *RAD51D* variants. Individualized modifications to the recommendations were applied especially in cases with complex genotypes comprising multiple (likely) pathogenic variants. In conclusion, this study demonstrates considerable heterogeneity in germline variants among individuals evaluated for HBOC susceptibility and highlights the particular challenges associated with interpreting moderate-risk gene alterations. The findings underscore the importance of individualized clinical assessment and support the use of real-world data to inform evidence-based surveillance and prevention strategies in hereditary cancer care.

**Supplementary Information:**

The online version contains supplementary material available at https://doi.org/10.1007/s10689-026-00609-5.

## Background

Hereditary breast and ovarian cancer (HBOC) syndrome is defined by an inherited susceptibility to breast and ovarian cancers and may further confer an increased risk of other malignancies, like pancreas or prostate cancer [1]. Pathogenic variants in HBOC risk genes are estimated to underlie 5 to 10% of breast cancer (BC) and at least 10 to 20% of ovarian cancer cases [2–6]. While genetic alterations of high-penetrance genes such as *BRCA1* and *BRCA2* remain the predominant contributors to HBOC, advances in multigene panel testing revealed a broad spectrum of genes implicated in HBOC predisposition [1, 2, 7]. Moderate-risk genes, including *ATM*, *BARD1*, *CHEK2*, *RAD51C*, and *RAD51D*, emerged as clinically relevant determinants to cancer susceptibility [8, 9]. These genes confer a lower but nevertheless meaningful elevation in lifetime cancer risk and are now routinely included in contemporary testing panels [8, 9].

Germline mutations in *ATM*, *BARD1*, and *CHEK2* are generally associated with a moderately increased lifetime risk of BC, estimated at approximately 20 to 30%, although specific genetic variants may confer higher or lower risks [9–16]. However, for carriers of *ATM*, *BARD1* and *CHEK2* pathogenic variants, there is no evidence supporting a predisposition to gynecologic malignancies [9, 12]. By contrast, *RAD51C* and *RAD51D* mutations are predominantly associated with heightened susceptibility to tubo-ovarian carcinoma (lifetime risk around 10%), while likewise conferring a moderate elevation in BC risk (lifetime risk around 10–30%) [9, 16, 17].

Despite widespread implementation of multigene panel testing, clinical management for carriers of moderate-risk pathogenic variants remains less clearly defined than for *BRCA1*/*BRCA2* carriers. In Germany, individuals carrying pathogenic variants in *ATM*, *BARD1*, *CHEK2*, *RAD51C*, and *RAD51D* are eligible for the intensified breast cancer surveillance (IBCS) program, with annual breast magnetic resonance imaging (MRI) as the central screening element [8]. Risk-reducing bilateral mastectomy (RRBM), however, is generally reserved for individuals with high-risk germline mutations, while ensuring that patient-specific risk factors are fully considered [8, 9]. Given the lack of effective screening for ovarian cancer and the concomitant elevation in ovarian cancer risk, risk-reducing salpingo-oophorectomy (RRSO) should be discussed with individuals carrying a *RAD51C* or *RAD51D* germline mutation upon completion of childbearing, typically around the onset of menopause [8, 9, 18]. It should be noted that guidance varies across international societies, and evidence is often extrapolated from retrospective analyses, small cohorts, or studies with heterogeneous designs [2, 7]. As a result, the identification of moderate-risk variants has become a challenging outcome of germline diagnostics in both predictive and disease-associated testing contexts. This uncertainty underscores the need for real-world data from specialized centers, where high testing volumes and structured genetic counseling pathways allow systematic evaluation of variant prevalence, phenotypic patterns, and clinical decision-making.

The HBOC center Erlangen (University Hospital Erlangen) is a member of the German Consortium for Hereditary Breast and Ovarian Cancer (GC-HBOC) and represents a certified center for HBOC risk assessment, offering comprehensive germline testing and long-term clinical management. The present study provides a seven-year real-world analysis of genetic findings from individuals who underwent predictive testing or were assessed in the context of breast malignancies or ovarian, fallopian tube, or primary peritoneal neoplasms at the HBOC center Erlangen from 2018 to 2024. With a particular focus on moderate-risk genes, we examined the frequency and distribution of genetic variants, their association with clinical characteristics, and the management strategies applied in routine care. By integrating genetic results with preventive strategies, this work aims to contribute to a more nuanced understanding of moderate-risk variant carriers and to support evidence-based approaches to personalized risk management in HBOC.

## Methods and materials

### Setting

The HBOC center Erlangen was established at the Department of Gynecology and Obstetrics of the University Hospital Erlangen in 2004. It joined the GC-HBOC in 2019 and was first certified by the German Cancer Society (Deutsche Krebsgesellschaft e.V., DKG) for the year 2021. Since certification, annual re-audits have ensured the continuous maintenance of high-quality standards in accordance with the requirements for HBOC-specialized centers.

### Patient cohort

From January 1, 2018 to December 31, 2024, a total of 3416 individuals were assessed at the HBOC center at University Hospital Erlangen. The cohort comprised both unaffected and affected persons, with indications for germline testing spanning preventive, diagnostic and therapeutic contexts. Individuals were classified as “unaffected” if they had no personal history of invasive BC or ductal carcinoma in situ (DCIS), ovarian, fallopian tube, or primary peritoneal cancer, borderline ovarian tumor (BOT), or serous tubal intraepithelial carcinoma (STIC) at the time of genetic testing. This group included both individuals undergoing predictive testing because of a known pathogenic germline variant in the family and individuals undergoing genetic testing based on a strong family history suggestive of hereditary cancer predisposition. Eligibility for genetic counseling was based on the criteria established by the German Cancer Society, which are systematically reviewed and updated regularly to incorporate the latest scientific evidence, resulting in periodic modifications during the study period. Current testing indications include multiple BC cases within a single parental lineage (at least three affected women or at least two affected women with at least one diagnosed before age 51), early-onset BC (diagnosed before age 36), bilateral BC (with the first diagnosis occurring before age 51), triple-negative BC before age 70, and combinations of breast and ovarian cancer within the family. Male BC and ovarian cancer diagnosed before age 80 also qualified. DCIS, STIC, and BOT were regarded as equivalent to invasive breast or ovarian cancer for the purpose of eligibility. The decision to include BOT was informed by the findings of Lhotova et al., who identified pathogenic germline variants in established cancer predisposition genes in a clinically relevant subset of patients with sporadic BOT [19]. Therapeutic germline testing was indicated following the approval of PARP (poly-(ADP-ribose)-polymerase) inhibitors for HER2 (human epidermal growth factor receptor 2)-negative BC. This applies to metastatic disease, and, in case of early BC, to HR (hormone receptor)-positive tumors with at least 4 positive lymph nodes or CPS-EG (Clinical Pathologic Stage - Estrogen Receptor Status and Grade) score ≥ 3 as well as to triple-negative tumors with non-pCR after neoadjuvant therapy [20–24].

### Genetic assessment workflow

Structured screening based on the current criteria of the German Cancer Society was used to determine eligibility for genetic testing. Individuals who fulfilled the requirements were invited to undergo genetic counseling at the HBOC center which was conducted by trained healthcare professionals with GC-HBOC basic module certification and additional qualifications in specialized human genetics counseling. After comprehensive counseling and sufficient time to consider, participants who provided written informed consent proceeded with genetic testing. For this purpose, next-generation sequencing (NGS) was performed on collected blood samples using a gene panel covering both high- and moderate-risk genes for breast and ovarian cancer. The panel composition, including updates over time based on current evidence, is shown in Table S1. Detected genetic variants were classified following the Human Genome Variation Society (HGVS) nomenclature. Patients were informed of the genetic results and their clinical implications. Depending on the genetic findings, clinical constellation and the patient’s wishes, IBCS or risk-reducing surgery was initiated where appropriate. Counseling outcomes were systematically documented.

### Clinical management of genetic variants

For (likely) pathogenic variants (PV; American College of Medical Genetics and Genomics (ACMG)/ International Agency for Research on Cancer (IARC) class 4 and 5), consideration is given to risk-reducing surgery (e.g., mastectomy, adnexectomy) or enrollment in IBCS, depending on the specific gene involved. Variants of uncertain significance (ACMGIARC class 3) are re-evaluated after three years, without immediate clinical intervention, while non-pathogenic results carry no direct clinical implications. Certain patient groups may still be eligible for preventive measures despite negative or uncertain genetic test results; for example, at the time of data collection, BC patients in Germany diagnosed before age 45 were eligible to participate in the IBCS program until age 50, irrespective of genetic findings.

### Data management and analysis

Patient data from the period between January 1, 2018, and December 31, 2024, at University Hospital Erlangen were collected retrospectively. Mutation frequencies in moderate-risk genes *ATM*, *BARD1*, *CHEK2*, *RAD51C*, and *RAD51D* were analyzed across all clinical subgroups irrespective of the gene’s predominant tumor association to allow for unbiased estimation of mutation frequencies within each cohort. For contextual interpretation, mutation frequencies of *BRCA1* and *BRCA2* were additionally assessed as high-penetrance reference genes.

Medical histories and results of human genetic testing were documented in an electronic case report form (eCRF) using Microsoft Access 365 version 2108. Figures were generated using Microsoft Excel version 2108.

## Results

### Patient characteristics

Between January 1, 2018, and December 31, 2024, a total of 3416 individuals underwent genetic testing at the HBOC center of the University Hospital Erlangen. Of these, 2397 (70.2%) were affected by cancer or precancerous lesions, 953 (27.9%) were unaffected and for 66 individuals (1.9%), it was not documented whether testing was performed due to a diagnosed condition. Of the total cohort, 3221 (94.3%) were female (see Table 1). Among affected individuals 98.9% were female, whereas the proportion of women among unaffected individuals was 82.8%. Age distribution showed that the largest group consisted of individuals aged 50–69 years (*n* = 1594, 46.7%), particularly among affected individuals, where this age group accounted for over half of those tested. In contrast, the age distribution among unaffected individuals was more balanced, with younger patients under 35 years representing a substantially larger proportion (*n* = 297, 31.2%) compared to affected individuals (*n* = 144, 6.0%).

**Table 1:** Patient demographics

Total number of tests	Affected individuals	Unaffected individuals	No information available	Total
	2397	953	66	3416
*Gender*				
Female	2371 (98.9%)	789 (82.8%)	61 (92.4%)	3221 (94.3%)
Male	26 (1.1%)	164 (17.2%)	5 (7.6%)	195 (5.7%)
*Age*				
< 35 years	144 (6.0%)	297 (31.2%)	7 (10.6%)	448 (13.1%)
≥ 35 years, < 50 years	656 (27.4%)	302 (31.7%)	8 (12.1%)	966 (28.3%)
≥ 50 years, < 70 years	1231 (51.4%)	317 (33.3%)	46 (69.7%)	1594 (46.7%)
≥ 70 years	366 (15.3%)	37 (3.9%)	5 (7.6%)	408 (11.9%)
Mean (median) age in years	55.3 (56)	44.2 (42)	55.4 (57)	52.2 (53)

Within the subgroup of affected individuals, 2032 presented with breast lesions and 414 with ovarian, fallopian tube, or primary peritoneal neoplasms as the indication for genetic testing (see Table 2). A number of patients fulfilled more than one diagnostic criterion, thereby contributing multiple qualifying conditions. When examining the distribution of diagnoses across the cohort, 1924 had a diagnosis of BC and 174 had precancerous breast lesions. Regarding gynecologic neoplasms, ovarian, fallopian tube, and primary peritoneal cancers constituted the largest group (*n* = 350), while BOT (*n* = 62) and STICs (*n* = 4) were less frequent. Furthermore, 62 individuals entered the testing cohort owing to a diagnosis of BOT

**Table 2:** Distribution of diagnoses among affected individuals (multiple diagnoses per individual possible)

Diagnosis	Affected individuals
Malignant breast disease	2032
BC	1924 (94.7%)
DCIS	174 (8.6%)
Ovarian, fallopian tube, and primary peritoneal neoplasms	414
Ovarian/ fallopian tube/ primary peritoneal cancer	350 (84.5%)
STIC	4 (1.0%)
BOT	62 (15.0%)

### Genetic findings

#### Genetic findings in affected individuals

**Fig. 1:**
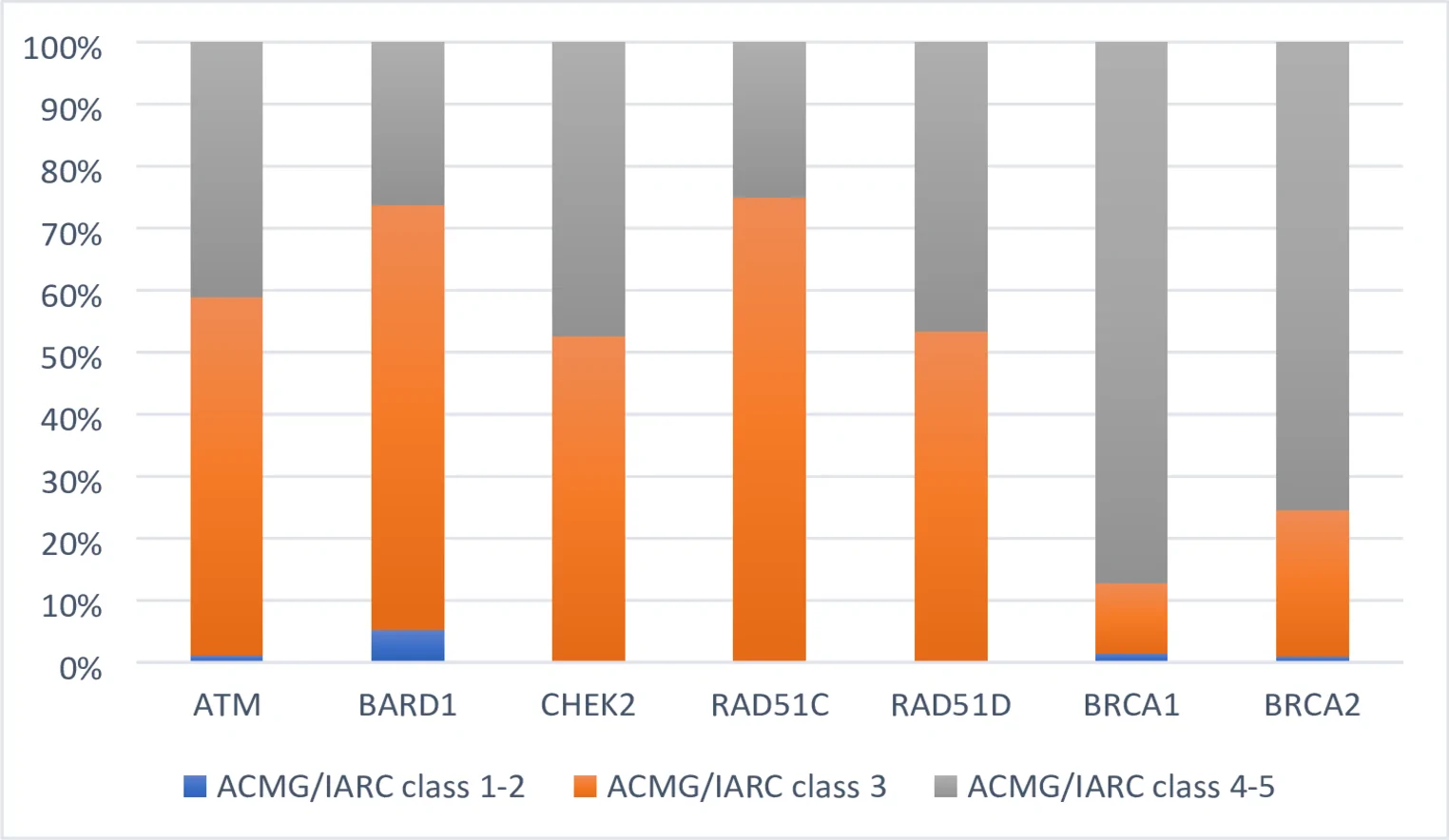
Distribution of genetic variants in patients with BC/ DCIS

Among patients undergoing germline testing for breast lesions, alterations in high-penetrance risk genes *BRCA1* and *BRCA2* were most frequently identified, as expected, with a total of 243 variants, including 199 PV. Focusing on moderate-risk genes for BC, numerous genetic alterations were likewise observed, albeit with a comparatively higher proportion of VUS. Among the moderate-risk genes, *CHEK2* exhibited the highest number of alterations with a total of 114 genetic variants, 54 (47.4%) of which were PV. Closely following was *ATM*, with 95 genetic variants, including 39 PV (41.1%). In contrast, the genes *BARD1*, *RAD51C*, and *RAD51D* showed markedly fewer genetic alterations in the cohort (in total 19, 8 and 15 genetic variants), the majority of which were classified as VUS (variant(s) of uncertain significance). The distribution of genetic variants in moderate-risk genes in the cohort with BC and/ or DCIS is presented in Table 3; Fig. 1, with *BRCA* genes included for comparison.

**Table 3:** Distribution of genetic variants stratified by disease status

Affected gene	Total	ACMG/IARC class 1–2	ACMG/IARC class 3	ACMG/IARC class 4–5	No ACMG/IARC class available
Patients with BC/ DCIS					
*ATM*	95	1 (1.1%)	55 (57.9%)	39 (41.1%)	0
*BARD1*	19	1 (5.3%)	13 (68.4%)	5 (26.3%)	0
*CHEK2*	114	0	60 (52.6%)	54 (47.4%)	0
*RAD51C*	8	0	6 (75.0%)	2 (25.0%)	0
*RAD51D*	15	0	8 (53.3%)	7 (46.7%)	0
*BRCA1*	133	2 (1.5%)	15 (11.3%)	116 (87.2%)	0
*BRCA2*	110	1 (0.9%)	26 (23.6%)	83 (75.5%)	0
Patients with neoplasms of the ovary, fallopian tube, and primary peritoneum					
*ATM*	8	0	3 (37.5%)	5 (62.5%)	0
*BARD1*	1	0	1 (100.0%)	0	0
*CHEK2*	6	0	5 (83.3%)	1 (16.7%)	0
*RAD51C*	1	0	1 (100.0%)	0	0
*RAD51D*	1	0	1 (100.0%)	0	0
*BRCA1*	42	0	2 (4.8%)	40 (95.2%)	0
*BRCA2*	24	0	3 (12.5%)	21 (87.5%)	0
Unaffected individuals					
*ATM*	21	0	12 (57.1%)	9 (42.9%)	0
*BARD1*	4	0	2 (50.0%)	2 (50.0%)	0
*CHEK2*	40	0	12 (30.0%)	28 (70.0%)	0
*RAD51C*	5	0	1 (20.0%)	4 (80.0%)	0
*RAD51D*	6	0	2 (33.3%)	4 (66.7%)	0
*BRCA1*	98	3 (3.1%)	2 (2.0%)	92 (93.9%)	1 (1.0%)
*BRCA2*	114	1 (0.9%)	5 (4.4%)	106 (93.0%)	2 (1.8%)

**Fig. 2:**
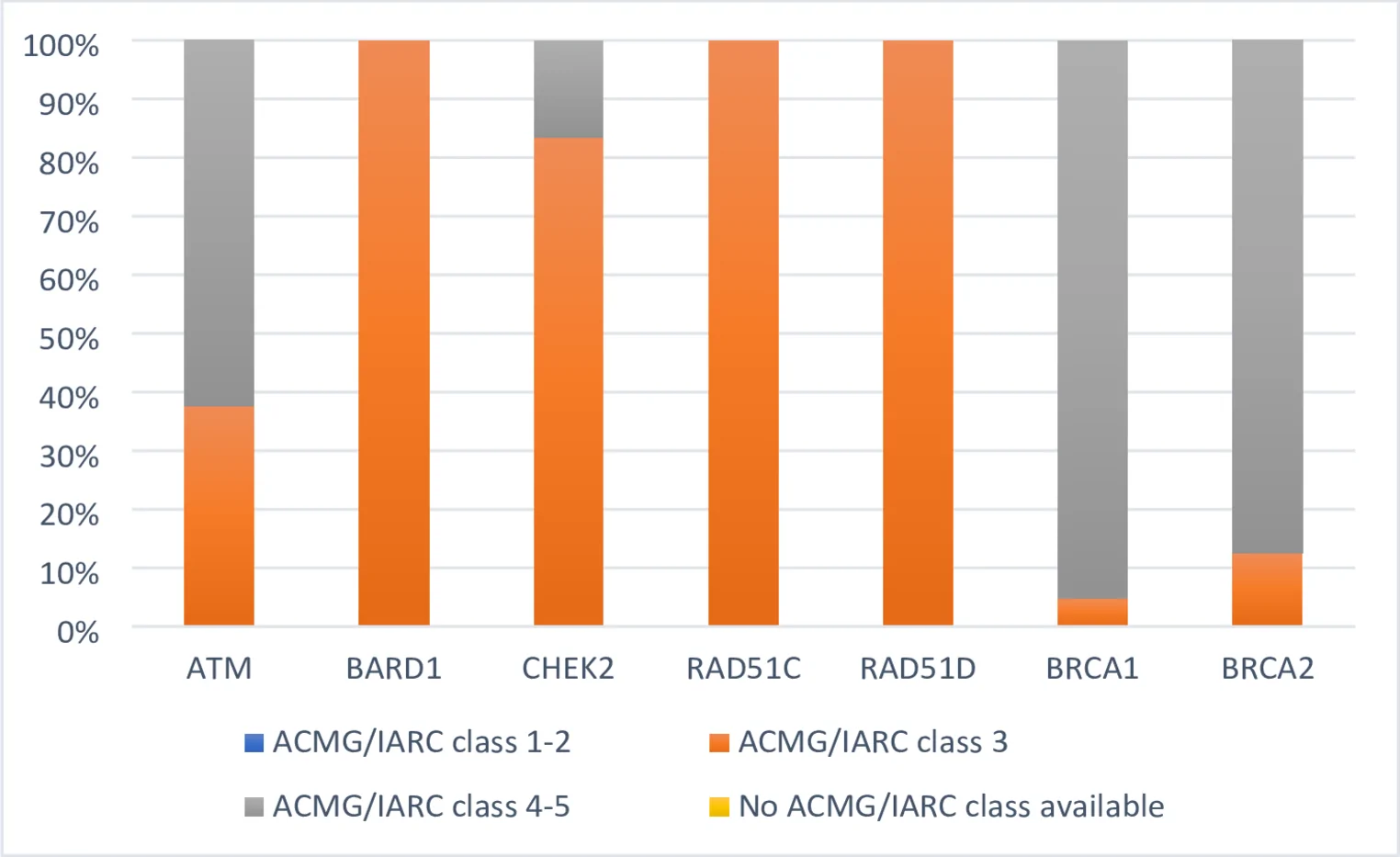
Distribution of genetic variants in patients with neoplasms of the ovary, fallopian tube, and primary peritoneum

In the subgroup of affected individuals with ovarian, fallopian tube, or primary peritoneal neoplasms, *BRCA1* and *BRCA2* once more represented the principal genes affected, with 66 detected variants, of which 61 were assigned to ACMG/ IARC classes 4 or 5 (see Table 3; Fig. 2). The moderate-risk BC genes *ATM* and *CHEK2* exhibited 8 and 6 genetic alterations, respectively, comprising a total of 6 PV. In contrast, the genes *RAD51C* and *RAD51D*, known to confer a moderate risk for BC but primarily associated with increased tubo-ovarian cancer risk, each harbored a single VUS. Likewise, one VUS was identified in *BARD1* (see Table 3; Fig. 2).

Within the STIC and BOT subgroups, we additionally assessed the distribution of variants in the moderate-risk BC genes investigated in this study. None of the four patients with STIC harbored a PV or VUS in these genes. Among the 62 patients with BOT, one patient (1.6%) harbored a PV in *CHEK2*, while three patients (4.8%) carried a VUS in *ATM*, *CHEK2*, and *BARD1*, respectively. In addition, no pathogenic *BRCA1* or *BRCA2* variants were identified in either subgroup.

#### Genetic findings in unaffected individuals

**Fig. 3:**
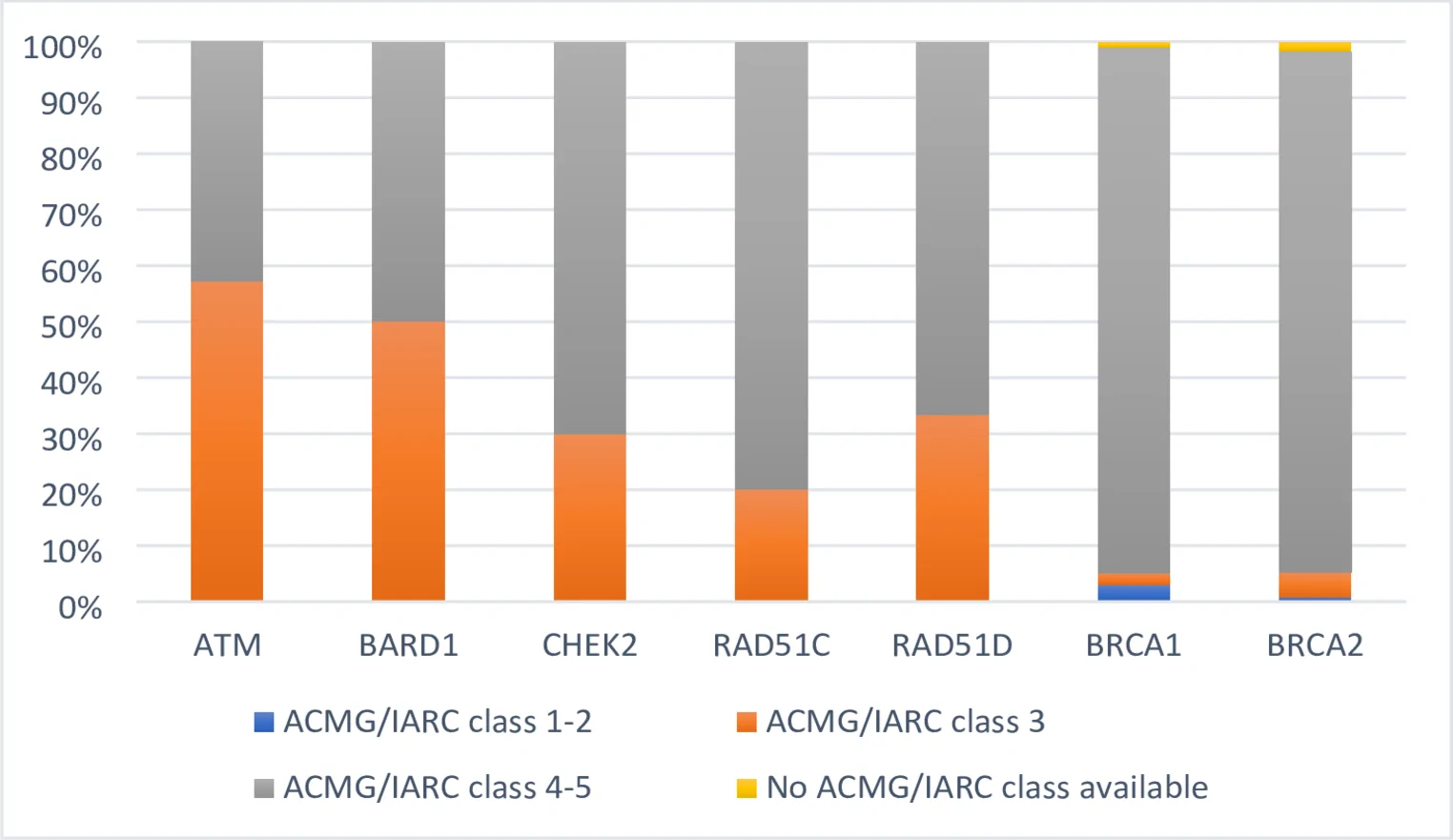
Distribution of genetic variants in unaffected individuals

In the cohort of unaffected individuals, the pattern of genetic alterations mirrors that seen in affected individuals, with *BRCA*1 and *BRCA2* again representing the most frequently affected genes (*BRCA1*: 98 variants (92 PV); *BRCA2*: 114 variants (106 PV), see Table 3; Fig. 3). Among the moderate-risk BC genes, 40 *CHEK2* variants were observed, 28 of which were PV (70.0%); 21 *ATM* variants, including 9 PV (42.9%); 5 *RAD51C* and 6 *RAD51D* variants, of which 4 in each gene were PV (80.0 and 66.7%, respectively); and 4 alterations in *BARD1*, including 2 PV (50.0%).

In the subgroup of tested individuals without available disease status (*n* = 66), PV were mainly detected in *BRCA1* (*n* = 5) and *BRCA2* (*n* = 6), while only few PV were identified in moderate-risk genes, including *CHEK2* (*n* = 1) and *RAD51C* (*n* = 1).

Detailed information on all identified variants in moderate-risk genes, including ACMG/IARC classification, coding and protein changes, zygosity, and frequency, is provided in Table S2.

### Moderate-risk variants and their clinical implications

#### *ATM* pathogenic variants

In the overall cohort of individuals tested at the HBOC center Erlangen between 2018 and 2024, 53 patients were identified as carriers of an *ATM* PV. The largest subgroup consisted of individuals with BC or DCIS (*n* = 39; see Table 4). Of the 39 *ATM* PV carriers with BC/DCIS, 37 had invasive BC, including two with an additional history of DCIS, while two had DCIS without invasive disease. The invasive tumors were predominantly HR-positive/HER2-negative (22/37, 59.5%), followed by HER2-positive (6/37, 16.2%) and triple-negative tumors (4/37, 10.8%). Tumor subtype could not be fully determined in the remaining five cases (13.5%) due to incomplete receptor data; four were HR-positive with unknown HER2 status, while both HR and HER2 status were unknown in one case. Five *ATM* PV carriers had ovarian or fallopian tube cancer: four had ovarian cancer (two serous, one clear-cell, and one endometrioid) and one had serous fallopian tube cancer. No primary peritoneal carcinoma, BOT, or STIC was observed in this subgroup. Of all *ATM* PV carriers, 50 (94.3%) were female. Among predictively tested women, all uniformly received a recommendation to participate in the IBCS program. Male carriers, for whom IBCS is not offered within the German healthcare system, accordingly did not receive such a recommendation. Among affected individuals, the majority were likewise advised to undergo IBCS (64.1% (25/39) of those with BC/DCIS; 60.0% (3/5) of those with ovarian, fallopian tube, or primary peritoneal neoplasms). Reasons for withholding an IBCS recommendation among BC/DCIS patients included metastatic disease (7/14, 50.0%), age-related considerations (3/14, 21.4%), and prior bilateral mastectomy for bilateral breast cancer (3/14, 21.4%); in one patient (7.1%), the reason was not documented. Only one patient, who additionally carried a *BRCA1* PV, was advised to consider RRBM. Three patients with an *ATM* PV were identified as candidates for RRSO; all three also harbored an additional PV in an ovarian cancer susceptibility gene, specifically *BRIP1*, *BRCA1*, or *BRCA2*.

#### *BARD1* pathogenic variants

Among the 7 individuals identified with a *BARD1* PV, 2 underwent predictive testing, whereas 5 were diagnosed with malignant breast disease (see Table 4). Notably, triple-negative BC (TNBC) was the predominant tumor subtype in *BARD1* PV carriers, accounting for four of five BC cases (80.0%), while one patient (20.0%) had HR-positive/HER2-negative BC. No HER2-positive tumors or isolated DCIS were observed. Furthermore, none of the *BARD1* PV carriers had an ovarian, fallopian tube, or primary peritoneal neoplasm. All *BARD1* PV carriers were female. None were considered candidates for prophylactic surgery. Participation in the IBCS program was recommended for nearly all carriers (85.7%); only one patient, who presented with metastatic BC at the time of evaluation, did not receive an IBCS recommendation.

#### *CHEK2* pathogenic variants

Across the entire cohort, a total of 83 *CHEK2* PV were identified in 82 individuals between 2018 and 2024. Among these individuals, 53 had BC or DCIS, including one person who carried two *CHEK2* PV and one who additionally had a history of BOT. Of the 53 *CHEK2* PV carriers with BC/DCIS, 52 had invasive BC, two of whom also had a history of DCIS, while one had DCIS without invasive disease. The invasive breast tumors were predominantly HR-positive/HER2-negative (26/52, 50.0%), followed by HER2-positive (12/52, 23.1%) and triple-negative tumors (4/52, 7.7%). Tumor subtype could not be fully determined in nine individuals (17.3%) due to incomplete receptor data; five had HR-positive tumors with unknown HER2 status, while both HR and HER2 status were unknown in four. One individual (1/52, 1.9%) had bilateral BC with discordant receptor profiles (HR-positive/HER2-negative on one side and HER2-positive on the contralateral side) and was therefore not assigned to a single tumor subtype category. This individual also had a history of BOT. No tubo-ovarian cancer, primary peritoneal carcinoma, or STIC was observed in the subgroup of *CHEK2* PV carriers. A further 28 individuals with *CHEK2* PV were unaffected, while disease status was unavailable for one individual.

The individual carrying two *CHEK2* PV had been diagnosed with BC and was advised to undergo IBCS or, alternatively, RRBM (see Table 4). Two additional BC patients, as well as one predictively tested woman, harbored a concurrent PV in a *BRCA* gene; accordingly, in addition to IBCS or the option of RRBM, RRSO was recommended. In all other cases, carriers of a *CHEK2* PV were uniformly not considered candidates for prophylactic surgery. Instead, the majority of women with a *CHEK2* PV were granted access to the IBCS program, comprising all predictively tested individuals and all individuals for whom disease status was unavailable, as well as 68.6% (35/51) of the women with a history of BC/DCIS, one of whom additionally had a history of BOT. Reasons for withholding an IBCS recommendation in this subgroup included metastatic disease (8/16, 50.0%), age-related considerations (5/16, 31.3%), prior bilateral mastectomy for bilateral breast disease (1/16, 6.3%), and severe comorbidities (1/16, 6.3%); in one case (6.3%), the reason remained unclear.

#### *RAD51C* and* RAD51D* pathogenic variants

Of the two affected *RAD51C* PV carriers, one had HR-positive/HER2-negative BC and one had isolated DCIS. Among the seven affected *RAD51D* PV carriers, three (42.9%) had TNBC, two (28.6%) HR-positive/HER2-negative BC, one (14.3%) HER2-positive BC, and one (14.3%) isolated DCIS without invasive disease. All other individuals carrying *RAD51C *or *RAD51D *PV had undergone predictive testing.

Of the 7 individuals harboring a *RAD51C* PV and the 11 individuals with a *RAD51D* PV, nearly all were advised to participate in the IBCS program (94.4%) and to undergo RRSO (88.9%, see Table 4). The option of RRBM was discussed only with one predictively tested woman who, in addition to a *RAD51D* PV, also carried a *BRCA2* PV. In another predictively tested individual, both a *RAD51C* PV and a *BRIP1* PV were identified.

#### Age distribution of PV carriers

As age represents an important factor in hereditary cancer risk assessment and may contribute to the clinical interpretation of PV, we further characterized PV carriers according to age at genetic testing and disease status. The distribution of PV carriers across these categories is presented in Table 5. *ATM* PV carriers were most frequently aged between 50 and 69 years (29/53, 54.7%), whereas *CHEK2* PV carriers were predominantly aged between 35 and 49 years (34/83, 41.0%) or between 50 and 69 years (31/83, 37.3%). Carrier numbers for *BARD1*, *RAD51C*, and *RAD51D* were substantially lower, limiting meaningful comparisons of age distributions across these genes.

**Table 4:** Clinical recommendations based on genetic findings

	Affected individuals			Unaffected individuals						No information available	
	BC/ DCIS		ovarian/ fallopian tube/ primary peritoneal neoplasm	family history of BC/ DCIS		family history of ovarian/ fallopian tube/ primary peritoneal neoplasm		family history of both, breast *and* ovarian/ fallopian tube/ primary peritoneal neoplasm			
	Female	Male	Female	Female	Male	Female	Male	Female	Male	Female	Male
*ATM* PV	39	0	5	4	2	0	0	2	1	0	0
IBCS	25	0	3	4	0	0	0	2	0	0	0
RRSO	3	0	0	0	0	0	0	0	0	0	0
RRBM	1	0	0	0	0	0	0	0	0	0	0
*BARD1* PV	5	0	0	2	0	0	0	0	0	0	0
IBCS	4	0	0	2	0	0	0	0	0	0	0
RRSO	0	0	0	0	0	0	0	0	0	0	0
RRBM	0	0	0	0	0	0	0	0	0	0	0
*CHEK2* PV	51	2	1	16	4	0	0	7	1	1	0
IBCS	35	0	1	16	0	0	0	7	0	1	0
RRSO	2	0	0	1	0	0	0	0	0	0	0
RRBM	3	0	0	1	0	0	0	0	0	0	0
*RAD51C* PV	2	0	0	1	0	0	0	3	0	1	0
IBCS	2	0	0	1	0	0	0	2	0	1	0
RRSO	2	0	0	1	0	0	0	2	0	1	0
RRBM	0	0	0	0	0	0	0	0	0	0	0
*RAD51D* PV	7	0	0	3	0	0	0	1	0	0	0
IBCS	7	0	0	3	0	0	0	1	0	0	0
RRSO	6	0	0	3	0	0	0	1	0	0	0
RRBM	0	0	0	0	0	0	0	1	0	0	0

BC = breast cancer. DCIS = ductal carcinoma in situ. PV = (likely) pathogenic variant(s). RRSO = risk-reducing bilateral salpingo-oophorectomy. RRBM = risk-reducing bilateral mastectomy

**Table 5:** Distribution of pathogenic variant carriers by age at genetic testing and disease status

Age at genetic testing	Affected individuals		Unaffected individuals	No information available	Total
	BC/DCIS	Ovarian/ fallopian tube/ primary peritoneal neoplasm			
*Individuals with ATM PV*					
< 35 years	0	1	4	0	5
≥ 35 years, < 50 years	12	0	3	0	15
≥ 50 years, < 70 years	25	2	2	0	29
≥ 70 years	2	2	0	0	4
*Individuals with BARD1 PV*					
< 35 years	0	0	1	0	1
≥ 35 years, < 50 years	2	0	1	0	3
≥ 50 years, < 70 years	3	0	0	0	3
≥ 70 years	0	0	0	0	0
*Individuals with CHEK2 PV*					
< 35 years	2	0	11	0	13
≥ 35 years, < 50 years	21	1	12	0	34
≥ 50 years, < 70 years	26	0	4	1	31
≥ 70 years	4	0	1	0	5
*Individuals with RAD51C PV*					
< 35 years	1	0	2	0	3
≥ 35 years, < 50 years	1	0	0	1	2
≥ 50 years, < 70 years	0	0	2	0	2
≥ 70 years	0	0	0	0	0
*Individuals with RAD51D*					
< 35 years	3	0	1	0	4
≥ 35 years, < 50 years	2	0	2	0	4
≥ 50 years, < 70 years	2	0	1	0	3
≥ 70 years	0	0	0	0	0

## Discussion

The present study provides a comprehensive overview of the spectrum and distribution pattern of germline variants identified in individuals who underwent predictive or disease-associated testing at the HBOC center Erlangen between 2018 and 2024. By evaluating a real-world cohort, this work offers important insights into the management of HBOC susceptibility in clinical practice. It focuses on moderate-risk gene alterations, whose interpretation is inherently more complex and less standardized than that of high-penetrance genes like *BRCA1* and *BRCA2*. Our data underscore that, in real-world clinical settings, recommendations for surveillance or risk-reducing interventions cannot always be applied uniformly. Instead, clinicians frequently take into account a variety of modifying factors, including the patient’s age, comorbidities, personal and family cancer history. These considerations may justify deviations from general guideline-based recommendations and reflect an increasingly personalized approach to hereditary cancer risk management.

Another notable finding is that, within the subgroup of moderate-risk variant carriers, several individuals were found to harbor more than one PV. The presence of multiple clinically relevant genetic alterations complicates risk assessment and highlights the importance of comprehensive panel testing. It further underscores the need for nuanced genetic counseling, as cumulative or interacting risks may influence both clinical decision-making and patient perceptions of their own risk. The recurrence of this phenomenon in our cohort emphasizes the real-world relevance of complex genotype constellations.

Of particular interest is the distribution of PV in *RAD51C* and *RAD51D*, genes primarily associated with an increased tubo-ovarian carcinoma risk and, at most, moderate BC susceptibility [9, 16, 17, 25]. In our cohort, such variants occurred exclusively among individuals undergoing predictive testing or those diagnosed with malignant breast disease. Strikingly, none were identified in individuals presenting with ovarian or other gynecologic malignancies. This observation appears unexpected in light of the well-established association of *RAD51C* and *RAD51D* PV with ovarian cancer (OC) risk and requires cautious interpretation, as it may arise from referral bias and from the limited size of the cohort. In a large analysis, Yang reported a relative risk of 7.60 (95% CI, 5.61–10.30) for tubo-ovarian cancer among carriers of PV in *RAD51C* or *RAD51D* [17]. These findings are further supported by the results of Suszynska et al. [26]. Nonetheless, it invites further investigation into genotype-phenotype correlations and potential modifiers of cancer risk in *RAD51C* and *RAD51D* variant carriers. Current evidence already suggests variability in OC risk of heterozygotes depending on the specific PV, familial disease burden, and additional genetic as well as non-genetic risk factors [25]. To improve individual risk assessment, the incorporation of a polygenic risk score (PRS) may be considered. Together with CanRisk, an online risk assessment tool developed at the University of Cambridge, the PRS can be integrated with genetic information and personal and family history to estimate an individual’s BC risk. Particularly in carriers of PV in moderate-risk genes, risk assessment can be assessed on an individual basis regarding to risk-reducing surgery [27]. Risk prediction for OC is currently still under development, and have not yet been established in routine clinical practice. Further validation is required. However, preliminary analyses have shown promising results, suggesting that the incorporation of PRS-based risk estimates may further improve individualized risk stratification in carriers of pathogenic variants in *RAD51C* and *RAD51D* [28]. This is particularly relevant because established guidelines, including GC-HBOC consensus recommendations, ESMO and NCCN guidelines, recommend consideration of RRSO for carriers of PV in *RAD51C* and *RAD51D* [8, 9, 18]. Although RRSO has been shown to effectively reduce the risk of tubo-ovarian cancer and no equivalent alternative preventive strategies for ovarian cancer are currently available, the indication for surgery should be carefully balanced against its potential harms [9, 25]. Reported long-term risks of premenopausal RRSO comprise osteoporosis, an increased cardiovascular risk as well as adverse effects on psychological and sexual wellbeing. Observational data further suggest a possible association with cognitive impairment [29–31]. Consequently, the timing of RRSO requires careful consideration, particularly in individuals with only moderately increased ovarian cancer risk. While ESMO and NCCN guidelines recommend the age of 45 to 50 years for RRSO in *RAD51C* and *RAD51D* PV carriers, the GC-HBOC consensus recommendations consider the onset of menopause an appropriate time point for risk-reducing surgery [8, 9, 18]. Given the phenotypic heterogeneity associated with moderate-risk genes and the influence of additional genetic and non-genetic modifying factors on the one hand, and the potentially far-reaching consequences of risk-reducing interventions on the other, large-scale studies of moderate-risk genes are needed to refine risk stratification and to support more individualized prevention strategies. Improved risk estimates may ultimately help to better guide clinical decision-making and to avoid both under- and overtreatment.

This study is subject to several limitations. As a single-center analysis, the findings may not be representative of broader populations. Genetic variant frequencies differ substantially across geographic and ethnic groups, and the size of the cohort imposes further constraints on generalizability. Nonetheless, the data offer valuable insights into real-world clinical practice, particularly regarding moderate-risk gene management, and may inform future multicenter efforts aimed at refining risk-adapted surveillance and prevention strategies.

## Conclusions

In summary, this study highlights the heterogeneity of germline findings among individuals assessed for HBOC susceptibility and the particular complexity of interpreting moderate-risk gene variants in routine clinical practice. The results underscore the necessity of personalized risk assessment, as management decisions should be tailored to individual risk factors. In this context, PRS may provide an additional tool to refine individual BC and OC risk estimates, particularly for carriers of PV in moderate-risk genes. More precise risk estimates may, in turn, support individualized genetic counseling and informed decision-making regarding risk-reducing strategies, including preventive surgery. As genetic testing becomes increasingly accessible, the integration of real-world data and multicenter studies will be essential to optimize evidence-based care for individuals at elevated hereditary cancer risk.

## Supplementary Information

Below is the link to the electronic supplementary material.

Supplementary Material 1

## Data Availability

The datasets used and analyzed during the current study are available upon reasonable request from the corresponding author.

## Abbreviations

ACMG: American College of Medical Genetics and Genomics
BC: breast cancer
BOT: borderline ovarian tumor(s)
DCIS: ductal carcinoma in situ
CPS-EG: Clinical Pathologic Stage - Estrogen Receptor status and grade
ESMO: European Society For Medical Oncology
GC-HBOC: German Consortium for Hereditary Breast and Ovarian Cancer
DKG: Deutsche Krebsgesellschaft e.V
eCRF: electronic case report form
HBOC: Hereditary Breast and Ovarian Cancer
HER2: human epidermal growth factor receptor 2
HGVS: Human Genome Variation Society
HR: hormone receptor
IARC: International Agency for Research on Cancer
IBCS: intensified breast cancer surveillance
MRI: magnetic resonance imaging
NCCN: National Comprehensive Cancer Network
NGS: next-generation sequencing
OC: Ovarian Cancer
PARP: poly-(ADP-ribose)-polymerase
PRS: polygenic risk score
PV: (likely) pathogenic variant(s)
RRBM: risk-reducing bilateral mastectomy
RRSO: risk-reducing bilateral salpingo-oophorectomy
STIC: serous tubal intraepithelial carcinoma
TNBC: triple-negative breast cancer
VUS: variant(s) of uncertain significance

## References

[1] Yoshida R (2021) Hereditary breast and ovarian cancer (HBOC): review of its molecular characteristics, screening, treatment, and prognosis. Breast Cancer 28(6):1167–1180 10.1007/s12282-020-01148-2 PMC8514387 32862296

[2] Marmolejo DH, Wong MYZ, Bajalica-Lagercrantz S, Tischkowitz M, Balmaña J, Patócs AB, Chappuis P, Colas C, Genuardi M, Haanpää M (2021) Overview of hereditary breast and ovarian cancer (HBOC) guidelines across Europe. Eur J Med Genet 64(12):104350 10.1016/j.ejmg.2021.104350 34606975

[3] Lux MP, Bani MR, Fasching PA, Beckmann MW (2016) Hereditary breast and ovarian cancer. Mol Basis Hum Cancer. 10.3238/arztebl.2011.0323

[4] Gupta S, Rajappa S, Advani S, Agarwal A, Aggarwal S, Goswami C, Palanki SD, Arya D, Patil S, Kodagali R (2021) Prevalence of BRCA1 and BRCA2 mutations among patients with ovarian, primary peritoneal, and fallopian tube cancer in India: a multicenter cross-sectional study. JCO Global Oncol 7:849–861 10.1200/GO.21.00051 PMC8457852 34101484

[5] Harter P, Hauke J, Heitz F, Reuss A, Kommoss S, Marmé F, Heimbach A, Prieske K, Richters L, Burges A (2017) Prevalence of deleterious germline variants in risk genes including BRCA1/2 in consecutive ovarian cancer patients (AGO-TR-1). PLoS ONE 12(10):e0186043 10.1371/journal.pone.0186043 PMC5650145 29053726

[6] Fasching PA, Yadav S, Hu C, Wunderle M, Haberle L, Hart SN, Rubner M, Polley EC, Lee KY, Gnanaolivu RD et al (2021) Mutations in BRCA1/2 and other panel genes in patients with metastatic breast cancer -association with patient and disease characteristics and effect on prognosis. J Clin Oncol 39(15):1619–1630 10.1200/JCO.20.01200 PMC8274805 33780288

[7] Kruckel A, Gocke J, Horner M, Keller K, Muller C, Bruckner L, Heindl F, Hack CC, Beckmann MW, Amann N (2026) Genetic tumor syndromes in female cancer: insights into inherited cancer predisposition and clinical implications. Arch Gynecol Obstet 313(1):38 10.1007/s00404-025-08270-6 PMC12799638 41528496

[8] Rhiem K, Auber B, Briest S, Dikow N, Ditsch N, Dragicevic N, Grill S, Hahnen E, Horvath J, Jaeger B (2022) Consensus recommendations of the German consortium for hereditary breast and ovarian cancer. Breast Care 17(2):199–207 10.1159/000516376 PMC9149395 35702495

[9] Sessa C, Balmaña J, Bober S, Cardoso M-J, Colombo N, Curigliano G, Domchek S, Evans D, Fischerova D, Harbeck N (2023) Risk reduction and screening of cancer in hereditary breast-ovarian cancer syndromes: ESMO Clinical Practice Guideline☆. Ann Oncol 34(1):33–47 10.1016/j.annonc.2022.10.004 36307055

[10] Thompson D, Duedal S, Kirner J, McGuffog L, Last J, Reiman A, Byrd P, Taylor M, Easton DF (2005) Cancer risks and mortality in heterozygous ATM mutation carriers. J Natl Cancer Inst 97(11):813–822 10.1093/jnci/dji141 15928302

[11] Jerzak K, Mancuso T, Eisen A (2018) Ataxia–telangiectasia gene (ATM) mutation heterozygosity in breast cancer: a narrative review. Curr Oncol 25(2):e176 10.3747/co.25.3707 PMC5927797 29719442

[12] Weber-Lassalle N, Borde J, Weber-Lassalle K, Horvath J, Niederacher D, Arnold N, Kaulfuss S, Ernst C, Paul VG, Honisch E et al (2019) Germline loss-of-function variants in the BARD1 gene are associated with early-onset familial breast cancer but not ovarian cancer. Breast Cancer Res 21(1):55 10.1186/s13058-019-1137-9 PMC6489184 31036035

[13] Slavin TP, Maxwell KN, Lilyquist J, Vijai J, Neuhausen SL, Hart SN, Ravichandran V, Thomas T, Maria A, Villano D et al (2017) The contribution of pathogenic variants in breast cancer susceptibility genes to familial breast cancer risk. NPJ Breast Cancer 3:22 10.1038/s41523-017-0024-8 PMC5466608 28649662

[14] Couch FJ, Shimelis H, Hu C, Hart SN, Polley EC, Na J, Hallberg E, Moore R, Thomas A, Lilyquist J (2017) Associations between cancer predisposition testing panel genes and breast cancer. JAMA Oncol 3(9):1190–1196 10.1001/jamaoncol.2017.0424 PMC5599323 28418444

[15] Cybulski C, Wokolorczyk D, Jakubowska A, Huzarski T, Byrski T, Gronwald J, Masojc B, Deebniak T, Gorski B, Blecharz P et al (2011) Risk of breast cancer in women with a CHEK2 mutation with and without a family history of breast cancer. J Clin Oncol 29(28):3747–3752 10.1200/JCO.2010.34.0778 21876083

[16] Breast Cancer Association C, Dorling L, Carvalho S, Allen J, Gonzalez-Neira A, Luccarini C, Wahlstrom C, Pooley KA, Parsons MT, Fortuno C et al (2021) Breast cancer risk genes - association analysis in more than 113,000 women. N Engl J Med 384(5):428–439 10.1056/NEJMoa1913948 PMC7611105 33471991

[17] Yang X, Song H, Leslie G, Engel C, Hahnen E, Auber B, Horvath J, Kast K, Niederacher D, Turnbull C et al (2020) Ovarian and breast cancer risks associated with pathogenic variants in RAD51C and RAD51D. J Natl Cancer Inst 112(12):1242–1250 10.1093/jnci/djaa030 PMC7735771 32107557

[18] Liu YL, Breen K, Catchings A, Ranganathan M, Latham A, Goldfrank DJ, Grisham RN, Long Roche K, Frey MK, Chi DS et al (2022) Risk-reducing bilateral salpingo-oophorectomy for ovarian cancer: a review and clinical guide for hereditary predisposition genes. JCO Oncol Pract 18(3):201–209 10.1200/OP.21.00382 PMC8932494 34582274

[19] Lhotova K, Stolarova L, Zemankova P, Vocka M, Janatova M, Borecka M, Cerna M, Jelinkova S, Kral J, Volkova Z et al (2020) Multigene panel germline testing of 1333 czech patients with ovarian cancer. Cancers (Basel). 10.3390/biomedicines8100404 PMC7226062 32295079

[20] Tutt ANJ, Garber JE, Kaufman B, Viale G, Fumagalli D, Rastogi P, Gelber RD, de Azambuja E, Fielding A, Balmana J et al (2021) Adjuvant olaparib for patients with BRCA1- or BRCA2-mutated breast cancer. N Engl J Med 384(25):2394–2405 10.1056/NEJMoa2105215 PMC9126186 34081848

[21] Robson M, Im SA, Senkus E, Xu B, Domchek SM, Masuda N, Delaloge S, Li W, Tung N, Armstrong A et al (2017) Olaparib for metastatic breast cancer in patients with a Germline BRCA mutation. N Engl J Med 377(6):523–533 10.1056/NEJMoa1706450 28578601

[22] Luftner D, Kolberg HC, Hartkopf AD, Fehm TN, Welslau M, Muller V, Schutz F, Fasching PA, Jackisch C, Marme F et al (2025) Update breast cancer 2024 part 3 - patients with advanced stage breast cancer. Geburtshilfe Frauenheilkd 85(5):507–519 10.1055/a-2515-2366 PMC12081098 40386501

[23] Park-Simon TW, Muller V, Albert US, Banys-Paluchowski M, Bartsch R, Bauerfeind I, Bjelic-Radisic V, Blohmer JU, Budach W, Dall P et al (2025) AGO recommendations for the diagnosis and treatment of patients with early breast cancer: Update 2025. Breast Care (Basel) 20(3):1–19 10.1159/000545019 PMC11996201 40236659

[24] Lukac S, Pfister K, Schaffler H, Leinert E, Fink A, Rack B, Fink V, Janni W, Heublein S (2025) Adjuvant targeted treatment of early hormone receptor-positive HER2-negative breast cancer: olaparib, abemaciclib or ribociclib - which one, how and for whom? Geburtshilfe Frauenheilkd 85(6):590–598 10.1055/a-2562-8469 PMC12158540 40510405

[25] Ngeow J, Chiang J, Astiazaran-Symonds E, Balmana J, Cass I, Kommoss FKF, Foulkes WD, James PA, Katcher A, Klugman S et al (2025) Management of individuals with heterozygous germline pathogenic variants in RAD51C, RAD51D, and BRIP1: A clinical practice resource of the American College of Medical Genetics and Genomics (ACMG). Genet Med 27(11):101557 10.1016/j.gim.2025.101557 PMC12862869 41070818

[26] Suszynska M, Ratajska M, Kozlowski P (2020) BRIP1, RAD51C, and RAD51D mutations are associated with high susceptibility to ovarian cancer: mutation prevalence and precise risk estimates based on a pooled analysis of ~ 30,000 cases. J Ovarian Res 13(1):50 10.1186/s13048-020-00654-3 PMC7196220 32359370

[27] Lee A, Mavaddat N, Cunningham A, Carver T, Ficorella L, Archer S, Walter FM, Tischkowitz M, Roberts J, Usher-Smith J et al (2022) Enhancing the BOADICEA cancer risk prediction model to incorporate new data on RAD51C, RAD51D, BARD1 updates to tumour pathology and cancer incidence. J Med Genet 59(12):1206–1218 10.1136/jmedgenet-2022-108471 PMC9691826 36162851

[28] Yang X, Wu Y, Ficorella L, Wilcox N, Dennis J, Tyrer J, Carver T, Pashayan N, Tischkowitz M, Pharoah PDP et al (2024) Validation of the BOADICEA model for epithelial tubo-ovarian cancer risk prediction in UK Biobank. Br J Cancer 131(9):1473–1479 10.1038/s41416-024-02851-z PMC11519606 39294438

[29] Faubion SS, Kuhle CL, Shuster LT, Rocca WA (2015) Long-term health consequences of premature or early menopause and considerations for management. Climacteric 18(4):483–491 10.3109/13697137.2015.1020484 PMC4581591 25845383

[30] Shuster LT, Gostout BS, Grossardt BR, Rocca WA (2008) Prophylactic oophorectomy in premenopausal women and long-term health. Menopause Int 14(3):111–116 10.1258/mi.2008.008016 PMC2585770 18714076

[31] Rocca WA, Gazzuola-Rocca L, Smith CY, Grossardt BR, Faubion SS, Shuster LT, Kirkland JL, Stewart EA, Miller VM (2016) Accelerated Accumulation of Multimorbidity After Bilateral Oophorectomy: A Population-Based Cohort Study. Mayo Clin Proc 91(11):1577–1589 10.1016/j.mayocp.2016.08.002 PMC5097693 27693001

