# Supplementary material for: Moderate-risk variants in hereditary breast and ovarian cancer: seven-year experience from a specialized center
Source: Fam Cancer. 2026 Oct 15;25(4):96. doi: 10.1007/s10689-026-00609-5 (PMC13672470; doi:10.1007/s10689-026-00609-5)
Supplement: Supplementary file 1 [file 10689_2026_609_MOESM1_ESM.docx]

**Supplementary material**

***Table S1.* Gene panel composition used at the HBOC center Erlangen, 2018-2024. X = gene included in the panel.**

***Table S2.*** **Genetic alterations in the cohort.**

|  | 2018 | 2019 | 2020 | 2021 | 2022 | 2023 | 2024 |
| --- | --- | --- | --- | --- | --- | --- | --- |
| BRCA1 | X | X | X | X | X | X | X |
| BRCA2 | X | X | X | X | X | X | X |
| CHEK2 | X | X | X | X | X | X | X |
| RAD51C | X | X | X | X | X | X | X |
| RAD51D | X | X | X | X | X | X | X |
| PALB2 | X | X | X | X | X | X | X |
| ATM | X | X | X | X | X | X | X |
| BRIP1 | X | X | X | X | X | X | X |
| CDH1 | X | X | X | X | X | X | X |
| TP53 | X | X | X | X | X | X | X |
| STK11 | X | X | X | X | X | X | X |
| PTEN | X | X | X | X | X | X | X |
| MLH1 | X | X | X | X | X | X | X |
| MSH2 | X | X | X | X | X | X | X |
| MSH6 | X | X | X | X | X | X | X |
| PMS2 | X | X | X | X | X | X | X |
| EPCAM |  |  |  |  | X | X | X |
| NBN | X |  |  |  |  |  |  |
| MRE11A | X |  |  |  |  |  |  |
| BARD1 | X |  |  | X | X | X | X |
| SMARCA4 |  |  |  |  | X | X | X |

*Table S1.* Gene panel composition used at the HBOC center Erlangen, 2018-2024. X = gene included in the panel.

| number | affected gene | ACMG/ IARC class | coding change | protein change | zygosity |
| --- | --- | --- | --- | --- | --- |
| 1 | *ATM* | 5 | c.824delT | p.(Leu275*) | heterozygous |
| 2 | *ATM* | 5 | c.3745A>T | p.(Arg1249*) | heterozygous |
| 3 | *ATM* | 5 | c.3868_3884del | p.(lle1290Serfs*6) | heterozygous |
| 4 | *ATM* | 5 | c.3712_3716del | p.(Leu1238Lysfs*6) | heterozygous |
| 5 | *ATM* | 5 | c.3802delG | p.(Val1268*) | heterozygous |
| 6 | *ATM* | 5 | c.7327C>T | p.(Arg2443*) | heterozygous |
| 7 | *ATM* | 5 | c.1524del | p.(Gly509Glufs*3) | heterozygous |
| 8 | *ATM* | 5 | c.4436G>A, r.4237_4436del | p.(Asp1413Alafs*11) | heterozygous |
| 9 | *ATM* | 5 | c.1402_1403del | p.(Lys468Glufs*18) | heterozygous |
| 10 | *ATM* | 5 | c.4436G>A | not available | heterozygous |
| 11 | *ATM* | 5 | c.8415_8418+4del | not available | heterozygous |
| 12 | *ATM* | 5 | c.2251-1G>C | not available | heterozygous |
| 13 | *ATM* | 5 | c.2441delA | p.(Asp814Alafs*9) | heterozygous |
| 14 | *ATM* | 5 | c.2441del | p.(Asp814Alafs*9) | heterozygous |
| 15 | *ATM* | 5 | c.3747-16A>G, r.3747_3903del | p.Ser1250ValfsTer47 | heterozygous |
| 16 | *ATM* | 5 | c.3802del | p.(Val1268Ter) | heterozygous |
| 17 | *ATM* | 5 | c.8147T>C | p.(Val2716Ala) | heterozygous |
| 18 | *ATM* | 5 | c.7886_7890del | p.(lle2629SerfsTer25) | heterozygous |
| 19 | *ATM* | 5 | c.902-1G>T | not available | heterozygous |
| 20 | *ATM* | 5 | c.790del | p.(Tyr264IIefsTer12) | heterozygous |
| 21 | *ATM* | 5 | c.1524del | p.(Gly509GlufsTer3) | heterozygous |
| 22 | *ATM* | 5 | c.7630-2A>C | not available | heterozygous |
| 23 | *ATM* | 5 | c.902-1G>T | not available | heterozygous |
| 24 | *ATM* | 5 | c.790del | p.(Tyr264IIefsTer12) | heterozygous |
| 25 | *ATM* | 5 | c.5932G>T | p.(Glu1978Ter) | heterozygous |
| 26 | *ATM* | 5 | c.2098C>T | p.(Gln700Ter) | heterozygous |
| 27 | *ATM* | 5 | c.7327C>T | p.(Arg2443Ter) | heterozygous |
| 28 | *ATM* | 5 | c.2441del | p.(Asp14AlafsTer9) | heterozygous |
| 29 | *ATM* | 5 | c.7630-2A>C | not available | heterozygous |
| 30 | *ATM* | 5 | c.7788G>A | p.(Glu2596=) | heterozygous |
| 31 | *ATM* | 5 | c.7327C>T | p.(Arg2443Ter) | heterozygous |
| 32 | *ATM* | 5 | c.790del | p.(Tyr264llefsTer12) | heterozygous |
| 33 | *ATM* | 5 | c.6095G>A | p.(Arg2032Lys) | heterozygous |
| 34 | *ATM* | 5 | c.7327C>T | p.(Arg2443Ter) | heterozygous |
| 35 | *ATM* | 5 | c.5932G>T | p.(Glu1978Ter) | heterozygous |
| 36 | *ATM* | 5 | c.790del | p.(Tyr264IlefsTer12) | heterozygous |
| 37 | *ATM* | 5 | c.5932G>T | p.(Glu1978Ter) | heterozygous |
| 38 | *ATM* | 5 | c.4777-10_4777-9delTC, r.4777_4909del | p.Glu1593IIefs*9 | heterozygous |
| 39 | *ATM* | 5 | c.2921+1G>A | not available | heterozygous |
| 40 | *ATM* | 5 | c.1524delT | p.(Gly509GlufsTer3) | heterozygous |
| 41 | *ATM* | 5 | c.3802del | p.(Val1268Ter) | heterozygous |
| 52 | *ATM* | 5 | c.3802del | p.(Val1268Ter) | heterozygous |
| 53 | *ATM* | 5 | c.8690del | p.(Gly2897AlafsTer41) | heterozygous |
| 42 | *ATM* | 4 | c.875C>T | p.(Pro292Leu) | heterozygous |
| 43 | *ATM* | 4 | c.8415_8418+4del | not available | heterozygous |
| 44 | *ATM* | 4 | c.2251-1G>C | not available | heterozygous |
| 45 | *ATM* | 4 | c.8147T>C | p.(Val2716Ala) | heterozygous |
| 46 | *ATM* | 4 | c.9023G>A | p.(Arg3008His) | heterozygous |
| 47 | *ATM* | 4 | c.3848T>C | p.(Leu1283Pro) | heterozygous |
| 48 | *ATM* | 4 | c.5497-2A>G | not available | heterozygous |
| 49 | *ATM* | 4 | c.875C>T | p.(Pro292Leu) | heterozygous |
| 50 | *ATM* | 4 | c.3747-1G>C | not available | heterozygous |
| 51 | *ATM* | 4 | c.8122G>A | p.(Asp2708Asn) | heterozygous |
| 54 | *ATM* | 3 | c.4777-10_4777-9delTC | not available | heterozygous |
| 55 | *ATM* | 3 | c.1340G>A | p.(Arg447Gln) | heterozygous |
| 56 | *ATM* | 3 | c.492G>T | p.(Trp164Cys) | heterozygous |
| 57 | *ATM* | 3 | c.61A>G | p.(Thr21Ala) | heterozygous |
| 58 | *ATM* | 3 | c.7475T>G | p.(Leu2492Arg) | heterozygous |
| 59 | *ATM* | 3 | c.8998C>G | p.(Gln3000Glu) | heterozygous |
| 60 | *ATM* | 3 | c.670A>G | p.(Lys224Glu) | heterozygous |
| 61 | *ATM* | 3 | c.6976-3C>T | not available | not available |
| 62 | *ATM* | 3 | c.7381C>T | p.(Arg2461Cys) | heterozygous |
| 63 | *ATM* | 3 | c.670A>G | p.(Lys224Glu) | heterozygous |
| 64 | *ATM* | 3 | c.5645G>A | p.(Arg1882Gln) | heterozygous |
| 65 | *ATM* | 3 | c.2110A>G | p.(Asn704Asp) | heterozygous |
| 66 | *ATM* | 3 | c.4612-18T>C | not available | heterozygous |
| 67 | *ATM* | 3 | c.5218A>G | p.(lle1740Val) | heterozygous |
| 68 | *ATM* | 3 | c.4577C>T | p.(Pro1526Leu) | heterozygous |
| 69 | *ATM* | 3 | c.670A>G | p.(Lys224Glu) | heterozygous |
| 70 | *ATM* | 3 | c.2420T>C | p.(Leu807Ser) | heterozygous |
| 71 | *ATM* | 3 | c.5494G>A | p.(Glu1832Lys) | heterozygous |
| 72 | *ATM* | 3 | c.7475T>G | p.(Leu2492Arg) | heterozygous |
| 73 | *ATM* | 3 | c.7457G>A | p.(Arg2486Gln) | heterozygous |
| 74 | *ATM* | 3 | c.9086G>A | p.(Gly3029Asp) | heterozygous |
| 75 | *ATM* | 3 | c.5262G>T | p.(Lys1754Asn) | heterozygous |
| 76 | *ATM* | 3 | c.3454T>C | p.(Ser1152Pro) | heterozygous |
| 77 | *ATM* | 3 | c.3145T>G | p.(Leu1049Val) | heterozygous |
| 78 | *ATM* | 3 | c.3676G>C | p.(Asp1226His) | heterozygous |
| 79 | *ATM* | 3 | c.9086G>A | p.(Gly3029Asp) | heterozygous |
| 80 | *ATM* | 3 | c.902G>A | p.(Gly301Asp) | heterozygous |
| 81 | *ATM* | 3 | c.6096-3T>C | not available | heterozygous |
| 82 | *ATM* | 3 | c.7475T>G | p.(Leu2492Arg) | heterozygous |
| 83 | *ATM* | 3 | c.1522C>T | p.(Leu508Phe) | heterozygous |
| 84 | *ATM* | 3 | c.6096-13_6096-9del | not available | heterozygous |
| 85 | *ATM* | 3 | c.7381C> T | p.(Arg2461Cys) | heterozygous |
| 86 | *ATM* | 3 | c.6343G>A | p.(Val2115IIe) | heterozygous |
| 87 | *ATM* | 3 | c.8264A>C | p.(Tyr2755Ser) | heterozygous |
| 88 | *ATM* | 3 | c.2353C>T | p.(Arg785Cys) | heterozygous |
| 89 | *ATM* | 3 | c.5418A>G | p.(lle1806Met) | heterozygous |
| 90 | *ATM* | 3 | c.8684A>C | p.(Glu2895Ala) | heterozygous |
| 91 | *ATM* | 3 | c.4082A>G | p.(Gln1361Arg) | heterozygous |
| 92 | *ATM* | 3 | c.5624G>A | p.(Arg1875Gln) | heterozygous |
| 93 | *ATM* | 3 | c.2681A>G | p.(Asp894Gly) | heterozygous |
| 94 | *ATM* | 3 | c.9155G>C | p.(Trp3052Ser) | heterozygous |
| 95 | *ATM* | 3 | c.3899A>G | p.(Tyr1300Cys) | heterozygous |
| 96 | *ATM* | 3 | c.7373A> T | p.(Asp2458Val) | heterozygous |
| 97 | *ATM* | 3 | c.2894A> T | p.(Asp965Val) | heterozygous |
| 98 | *ATM* | 3 | c.716T>C | p.(Phe239Ser) | heterozygous |
| 99 | *ATM* | 3 | c.100A>G | p.(lle34Val) | heterozygous |
| 100 | *ATM* | 3 | c.5866C>T | p.(Leu1956Phe) | heterozygous |
| 101 | *ATM* | 3 | c.1121A>G | p.(Gln37 4Arg) | heterozygous |
| 102 | *ATM* | 3 | c.7475T>G | p.(Leu2492Arg) | heterozygous |
| 103 | *ATM* | 3 | c.902G>A | p.(Gly301Asp) | heterozygous |
| 104 | *ATM* | 3 | c.8263T>C | p.(Tyr2755His) | heterozygous |
| 105 | *ATM* | 3 | c.8867C>T | p.(Pro2956Leu) | heterozygous |
| 106 | *ATM* | 3 | c.2062G>A | p.(Glu688Lys) | heterozygous |
| 107 | *ATM* | 3 | c.4421A>G | p.(His1474Arg) | heterozygous |
| 108 | *ATM* | 3 | c.9166G>T | p.(Val3056Leu) | heterozygous |
| 109 | *ATM* | 3 | c.9155G>C | p.(Trp3052Ser) | heterozygous |
| 110 | *ATM* | 3 | c.6323A>G | p.(Gln2108Arg) | heterozygous |
| 111 | *ATM* | 3 | c.4329C>A | p.(His1443Gln) | heterozygous |
| 112 | *ATM* | 3 | c.8734A>G | p.(Arg2912Gly) | heterozygous |
| 113 | *ATM* | 3 | c.8750G>A | p.(Gly291Asp) | heterozygous |
| 114 | *ATM* | 3 | c.6661G>A | p.(Glu2221Lys) | heterozygous |
| 115 | *ATM* | 3 | c.4468A>G | p.(Ser1490Gly) | heterozygous |
| 116 | *ATM* | 3 | c.3059C>T | p.(Thr1020Ile) | heterozygous |
| 117 | *ATM* | 3 | c.3256C>T | p.(Arg1086Cys) | heterozygous |
| 118 | *ATM* | 3 | c.4133C>T | p.(Pro1378Leu) | heterozygous |
| 119 | *ATM* | 3 | c.4304A>G | p.(Lys1435Arg) | heterozygous |
| 120 | *ATM* | 3 | c.6974C>T | p.(Ala2325Val) | heterozygous |
| 121 | *ATM* | 3 | c.8560C>T | p.(Arg2854Cys) | heterozygous |
| 122 | *ATM* | 3 | c.8734A>G | p.(Arg2912Gly) | heterozygous |
| 123 | *ATM* | 2 | c.8850+5A>C | not available | heterozygous |
| 124 | *BARD1* | 5 | c.1935_1954dup20 | p.(Glu652Valfs*69) | heterozygous |
| 125 | *BARD1* | 5 | c.2032delT | p.(Tyr678llefs*36) | heterozygous |
| 126 | *BARD1* | 4 | c.1876_1879del | p.(Gly627AspfsTer5) | heterozygous |
| 127 | *BARD1* | 4 | c.1876_1879del | p.(Gly627AspfsTer5) | heterozygous |
| 128 | *BARD1* | 4 | c.1932_1933del | p.(Val644_Cys645insTer) | heterozygous |
| 129 | *BARD1* | 4 | c.1811-2A>G | not available | heterozygous |
| 130 | *BARD1* | 4 | c.2300_2301del | p.(Val767AspfsTer4) | heterozygous |
| 131 | *BARD1* | 3 | c.1226C>G | p.(Ser409Cys) | heterozygous |
| 132 | *BARD1* | 3 | c.(?_-29)_(2334_ ?)dup | not available | heterozygous |
| 133 | *BARD1* | 3 | c.1835A>T | p.(Asp612Val) | heterozygous |
| 134 | *BARD1* | 3 | c.1858T>C | p.(Cys620Arg) | heterozygous |
| 135 | *BARD1* | 3 | c.26_40del | p.(Asn9_Arg13del) | heterozygous |
| 136 | *BARD1* | 3 | c.679G>A | p.(Gly227Ser) | heterozygous |
| 137 | *BARD1* | 3 | c.1988A>G | p.(Asn663Ser) | heterozygous |
| 138 | *BARD1* | 3 | c.1403C>T | p.(Ala468Val) | heterozygous |
| 139 | *BARD1* | 3 | c.127C>T | p.(Arg43Cys) | heterozygous |
| 140 | *BARD1* | 3 | c.233G>A | p.(Cys78Tyr) | heterozygous |
| 141 | *BARD1* | 3 | c.1693C>T | p.(Arg565Cys) | heterozygous |
| 142 | *BARD1* | 3 | c.433A>G | p.(Met145Val) | heterozygous |
| 143 | *BARD1* | 3 | c.1915T>C | p.(Cys639Arg) | heterozygous |
| 144 | *BARD1* | 3 | c.1333G>A | p.(Glu445Lys) | heterozygous |
| 145 | *BARD1* | 3 | c.2216A>G | p.(Tyr739Cys) | heterozygous |
| 146 | *BARD1* | 3 | c.1463A>G | p.(Asn488Ser) | heterozygous |
| 147 | *BARD1* | 2 | c.353A>G | p.(Asn118Ser) | heterozygous |
| 148 | *CHEK2* | 5 | c.(975+1_976-1)_(1037+1_1038-1)del | p.(Met304AspfsTer8) | heterozygous |
| 149 | *CHEK2* | 5 | c.592+3A>T | not available | not available |
| 150 | *CHEK2* | 5 | c.470T>C | p.(lle157Thr) | heterozygous |
| 151 | *CHEK2* | 5 | c.1038_1224del186 | p.(Met347Leufs*16) | heterozygous |
| 152 | *CHEK2* | 5 | c.432delT | p.(Arg145Glyfs*16) | heterozygous |
| 153 | *CHEK2* | 5 | c.432delT | p.(Arg145Glyfs*16) | heterozygous |
| 154 | *CHEK2* | 5 | c.1100delC | p.(Thr367Metfs*15) | heterozygous |
| 155 | *CHEK2* | 5 | c.1100delC | p.(Thr367Metfs*15) | heterozygous |
| 156 | *CHEK2* | 5 | c.1100delC | p.(Thr367Metfs*15) | heterozygous |
| 157 | *CHEK2* | 5 | c.1100delC | p.(Thr367Metfs*15) | heterozygous |
| 158 | *CHEK2* | 5 | c.1100delC | p.(Thr367Metfs*15) | heterozygous |
| 159 | *CHEK2* | 5 | c.1100delC | p.(Thr367Metfs*15) | heterozygous |
| 160 | *CHEK2* | 5 | c.1238T>G | p.(Leu413*) | heterozygous |
| 161 | *CHEK2* | 5 | c.1100delC | p.(Thr367Metfs*15) | heterozygous |
| 162 | *CHEK2* | 5 | c.349A>G | p.(Argll7Gly) | heterozygous |
| 163 | *CHEK2* | 5 | c.1100delC | p.(Thr367Metfs*15) | heterozygous |
| 164 | *CHEK2* | 5 | c.1100delC | p.(Thr367Metfs*15) | heterozygous |
| 165 | *CHEK2* | 5 | c.1100delC | p.(Thr367Metfs*15) | heterozygous |
| 166 | *CHEK2* | 5 | c.444+1G>A | not available | heterozygous |
| 167 | *CHEK2* | 5 | c.(908+1_909-1)_(1095+1_1096-1)del | p.(Met304Leufs*16) | heterozygous |
| 168 | *CHEK2* | 5 | c.478A>G | p.(Arg160Gly) | heterozygous |
| 169 | *CHEK2* | 5 | c.1100delC | p.(Thr367Metfs*15) | heterozygous |
| 170 | *CHEK2* | 5 | c.(908+1_909-1)_(1095+1_1096-1)del | p.(Met304LeufsTer16) | heterozygous |
| 171 | *CHEK2* | 5 | c.1100delC | p.(Thr367Metfs*15) | heterozygous |
| 172 | *CHEK2* | 5 | c.1100del | p.(Thr367MetfsTer15) | heterozygous |
| 173 | *CHEK2* | 5 | c.277del | p.(Trp93GlyfsTer44) | heterozygous |
| 174 | *CHEK2* | 5 | c.1100delC | p.(Thr367MetfsTer15) | heterozygous |
| 175 | *CHEK2* | 5 | c.[(908+1_909-1)_(1095+1_1096-1)del;c.1139_1140del] | p.[(Met304LeufsTer16;Leu380HisfsTer14)] | heterozygous |
| 176 | *CHEK2* | 5 | c.1100delC | p.(Thr367Metfs*15) | heterozygous |
| 177 | *CHEK2* | 5 | c.1100delC | p.(Thr367Metfs*15) | heterozygous |
| 178 | *CHEK2* | 5 | c.1100delC | p.(Thr367MetfsTer15) | heterozygous |
| 179 | *CHEK2* | 5 | c.1100del | p.(Thr367MetfsTer15) | heterozygous |
| 180 | *CHEK2* | 5 | c.444+1G>A | not available | heterozygous |
| 181 | *CHEK2* | 5 | c.(908+1_909-1)_(1095+1_1096-l)del | p.(Met304LeufsTer16) | heterozygous |
| 182 | *CHEK2* | 5 | c.277del | p.(Trp93GlyfsTer17) | heterozygous |
| 183 | *CHEK2* | 5 | c.444+1G>A | not available | heterozygous |
| 184 | *CHEK2* | 5 | c.660C>A | p.(Tyr220Ter) | heterozygous |
| 185 | *CHEK2* | 5 | c.591del | p.(Val198PhefsTer7) | heterozygous |
| 186 | *CHEK2* | 5 | c.1100del | p.(Thr357MetfsTer15) | heterozygous |
| 187 | *CHEK2* | 5 | c.1100del | p.(Thr367MetfsTer15) | heterozygous |
| 188 | *CHEK2* | 5 | c.(908+1_909-1)_(1095+1_1096-1)del | p.(Met304LeufsTer16) | heterozygous |
| 189 | *CHEK2* | 5 | c.1100delC | p.(Thr367MetfsTer15) | heterozygous |
| 190 | *CHEK2* | 5 | c.444+1G>T | not available | heterozygous |
| 191 | *CHEK2* | 5 | c.1100delC | p.(Thr367MetfsTer15) | heterozygous |
| 192 | *CHEK2* | 5 | c.592+3A>T | not available | heterozygous |
| 193 | *CHEK2* | 5 | c.592+3A>T | not available | heterozygous |
| 194 | *CHEK2* | 5 | c.444+1G>T | not available | heterozygous |
| 195 | *CHEK2* | 5 | c.339C>G | p.(Tyr113Ter) | heterozygous |
| 196 | *CHEK2* | 5 | c.1100delC | p.(Thr367MetfsTer15) | heterozygous |
| 197 | *CHEK2* | 5 | c.1100delC | p.(Thr367MetfsTer15) | heterozygous |
| 198 | *CHEK2* | 5 | c.592+3A>T | not available | heterozygous |
| 199 | *CHEK2* | 5 | c.592+3A>T | not available | heterozygous |
| 200 | *CHEK2* | 5 | c.(908+1_909-1)_(1095+1_1096-1)del | p.(Met304LeufsTer16) | heterozygous |
| 201 | *CHEK2* | 5 | c.1100delC | p.(Thr367MetfsTer15) | heterozygous |
| 202 | *CHEK2* | 5 | c.507del | p.(Phe169LeufsTer2) | heterozygous |
| 203 | *CHEK2* | 5 | c.1100del | p.(Thr367MetfsTer15) | heterozygous |
| 204 | *CHEK2* | 5 | c.507del | p.(Phe169LeufsTer2) | heterozygous |
| 205 | *CHEK2* | 5 | c.1100delC | p.(Thr367MetfsTer15) | heterozygous |
| 206 | *CHEK2* | 5 | c.1472C>T | p.(Thr476Met) | heterozygous |
| 207 | *CHEK2* | 5 | c.507del | p.(Phe169LeufsTer2) | heterozygous |
| 208 | *CHEK2* | 5 | c.507del | p.(Phe169LeufsTer2) | heterozygous |
| 209 | *CHEK2* | 5 | c.1100delC | p.(Thr367MetfsTer15) | heterozygous |
| 210 | *CHEK2* | 5 | c.1100delC | p.(Thr367MetfsTer15) | heterozygous |
| 211 | *CHEK2* | 5 | c.1100delC | p.(Thr367Metfs*15) | heterozygous |
| 212 | *CHEK2* | 5 | c.507del | p.(Phe212Leufs*2) | heterozygous |
| 213 | *CHEK2* | 5 | c.1100delC | p.(Thr367Metfs*15) | heterozygous |
| 214 | *CHEK2* | 5 | c.591del | p.(Val198PhefsTer7) | heterozygous |
| 215 | *CHEK2* | 5 | c.1100delC | p.(Thr367MetfsTer15) | heterozygous |
| 216 | *CHEK2* | 5 | c.1100del | p.(Thr367MetfsTer15) | heterozygous |
| 217 | *CHEK2* | 5 | c.1100delC | p.(Thr367Metfs*15) | heterozygous |
| 218 | *CHEK2* | 5 | c.1100delC | p.(Thr367Metfs*15) | heterozygous |
| 219 | *CHEK2* | 5 | c.(975+1_976-1)_(1037+1_1038-1)del | p.(Met304AspfsTer8) | heterozygous |
| 220 | *CHEK2* | 4 | c.499G>A | p.(Gly167Arg) | heterozygous |
| 221 | *CHEK2* | 4 | c.190G>A | p.(Glu64Lys) | heterozygous |
| 222 | *CHEK2* | 4 | c.433C>T | p.(Arg145Trp) | heterozygous |
| 223 | *CHEK2* | 4 | c.1039G>A | p.(Asp347Asn) | heterozygous |
| 224 | *CHEK2* | 4 | c.349A>G | p.(Arg117Gly) | heterozygous |
| 225 | *CHEK2* | 4 | c.349A>G | p.(Arg117Gly) | heterozygous |
| 226 | *CHEK2* | 4 | c.433C>T | p.(Arg145Trp) | heterozygous |
| 227 | *CHEK2* | 4 | c.1420C>T | p.(Arg474Cys) | heterozygous |
| 228 | *CHEK2* | 4 | c.1270T>C | p.(Tyr424His) | heterozygous |
| 229 | *CHEK2* | 4 | c.1169A>C | p.(Tyr390Ser) | heterozygous |
| 230 | *CHEK2* | 4 | c.349A>G | p.(Arg117Gly) | heterozygous |
| 231 | *CHEK2* | 3 | c.470T>C | p.(lle157Thr) | heterozygous |
| 232 | *CHEK2* | 3 | c.470T>C | p.(Ile157Thr) | heterozygous |
| 233 | *CHEK2* | 3 | c.470T>C | p.(lle157Thr) | heterozygous |
| 234 | *CHEK2* | 3 | c.320-5T>A | not available | heterozygous |
| 235 | *CHEK2* | 3 | c.715G>A | p.(Glu239Lys) | heterozygous |
| 236 | *CHEK2* | 3 | c.470T>C | p.(lle157Thr) | heterozygous |
| 237 | *CHEK2* | 3 | c.556A>C | p.(Asn186His) | heterozygous |
| 238 | *CHEK2* | 3 | c.1160C>G | p.(Thr387Ser) | heterozygous |
| 239 | *CHEK2* | 3 | c.1423T>A | p.(Phe475IIe) | heterozygous |
| 240 | *CHEK2* | 3 | c.470T>C | p.(lle157Thr) | heterozygous |
| 241 | *CHEK2* | 3 | c.470T>C | p.(lle157Thr) | heterozygous |
| 242 | *CHEK2* | 3 | c.524T>C | p.(Val175Ala) | heterozygous |
| 243 | *CHEK2* | 3 | c.1383C>G | p.(Asp461Glu) | heterozygous |
| 244 | *CHEK2* | 3 | c.470T>C | p.(lle157Thr) | heterozygous |
| 245 | *CHEK2* | 3 | c.442A>G | p.(Arg148Gly) | heterozygous |
| 246 | *CHEK2* | 3 | c.1091T>C | p.(lle364Thr) | heterozygous |
| 247 | *CHEK2* | 3 | c.470T>C | p.(lle157Thr) | heterozygous |
| 248 | *CHEK2* | 3 | c.422A>C | p.(Lys141Thr) | heterozygous |
| 249 | *CHEK2* | 3 | c.470T>C | p.(lle157Thr) | heterozygous |
| 250 | *CHEK2* | 3 | c.470T>C | p.(lle157Thr) | heterozygous |
| 251 | *CHEK2* | 3 | c.1036C>T | p.(Arg346Cys) | heterozygous |
| 252 | *CHEK2* | 3 | c.1567C>G | p.(Arg523Gly) | heterozygous |
| 253 | *CHEK2* | 3 | c.470T>C | p.(lle157Thr) | heterozygous |
| 254 | *CHEK2* | 3 | c.917G>C | p.(Gly306Ala) | heterozygous |
| 255 | *CHEK2* | 3 | c.470T>C | p.(lle157Thr) | heterozygous |
| 256 | *CHEK2* | 3 | c.470T>C | p.(lle157Thr) | heterozygous |
| 257 | *CHEK2* | 3 | c.470T>C | p.(lle157Thr) | heterozygous |
| 258 | *CHEK2* | 3 | c.1427C>T | p.(Thr476Met) | heterozygous |
| 259 | *CHEK2* | 3 | c.470T>C | p.(lle157Thr) | heterozygous |
| 260 | *CHEK2* | 3 | c.470T>C | p.(lle157Thr) | heterozygous |
| 261 | *CHEK2* | 3 | c.470T>C | p.(lle157Thr) | heterozygous |
| 262 | *CHEK2* | 3 | c.910A>G | p.(Met304Val) | heterozygous |
| 263 | *CHEK2* | 3 | c.470T>C | p.(lle157Thr) | heterozygous |
| 264 | *CHEK2* | 3 | c.1259G>A | p.(Cys420Tyr) | heterozygous |
| 265 | *CHEK2* | 3 | c.917G>C | p.(Gly306Ala) | heterozygous |
| 266 | *CHEK2* | 3 | c.470T>C | p.(lle157Thr) | heterozygous |
| 267 | *CHEK2* | 3 | g.(?_29083875)_(29085213_?)dup | not available | not available |
| 268 | *CHEK2* | 3 | c.470T>C | p.(lle157Thr) | heterozygous |
| 269 | *CHEK2* | 3 | c.524T>C | p.(Val175Ala) | heterozygous |
| 270 | *CHEK2* | 3 | c.556A>C | p.(Asn186His) | heterozygous |
| 271 | *CHEK2* | 3 | c.848C>A | p.(Pro283His) | heterozygous |
| 272 | *CHEK2* | 3 | c.661A>G | p.(lle221Val) | heterozygous |
| 273 | *CHEK2* | 3 | c.470T>C | p.(lle157Thr) | heterozygous |
| 274 | *CHEK2* | 3 | c.1274C>T | p.(Pro425Leu) | heterozygous |
| 275 | *CHEK2* | 3 | c.470T>C | p.(lle157Thr) | heterozygous |
| 276 | *CHEK2* | 3 | c.661A>G | p.(lle221Val) | heterozygous |
| 277 | *CHEK2* | 3 | c.146C>T | p.(Ser49Phe) | heterozygous |
| 278 | *CHEK2* | 3 | c.1427C>T | p.(Thr476Met) | heterozygous |
| 279 | *CHEK2* | 3 | c.1427C>T | p.(Thr476Met) | heterozygous |
| 280 | *CHEK2* | 3 | c.470T>C | p. lle157Thr) | heterozygous |
| 281 | *CHEK2* | 3 | c.470T>C | p.(lle157Thr) | heterozygous |
| 282 | *CHEK2* | 3 | c.422A>C | p.(Lys141 Thr) | heterozygous |
| 283 | *CHEK2* | 3 | c.470T>C | p.(lle157Thr) | heterozygous |
| 284 | *CHEK2* | 3 | c.1313A>G | p.(Asp438Gly) | heterozygous |
| 285 | *CHEK2* | 3 | c.779C>T | p.(Ser260Leu) | heterozygous |
| 286 | *CHEK2* | 3 | c.1451C>T | p.(Pro484Leu) | heterozygous |
| 287 | *CHEK2* | 3 | c.1175C> T | p.(Ala392Val) | heterozygous |
| 288 | *CHEK2* | 3 | c.246_260del | p.(Asp82_Glu86del) | heterozygous |
| 289 | *CHEK2* | 3 | c.578T>C | p.(Leu193Pro) | heterozygous |
| 290 | *CHEK2* | 3 | c.470T>C | p.(lle157Thr) | heterozygous |
| 291 | *CHEK2* | 3 | c.470T>C | p.(lle157Thr) | heterozygous |
| 292 | *CHEK2* | 3 | c.190G>A | p.(Glu64Lys) | heterozygous |
| 293 | *CHEK2* | 3 | c.470T>C | p.(lle157Thr) | heterozygous |
| 294 | *CHEK2* | 3 | c.470T>C | p.(lle157Thr) | heterozygous |
| 295 | *CHEK2* | 3 | c.470T>C | p.(lle157Thr) | heterozygous |
| 296 | *CHEK2* | 3 | c.1186C>G | p.(Leu396Val) | heterozygous |
| 297 | *CHEK2* | 3 | c.496A>G | p.(Asn166Asp) | heterozygous |
| 298 | *CHEK2* | 3 | c.1033C> T | p.(His345Tyr) | heterozygous |
| 299 | *CHEK2* | 3 | c.470T>C | p.(lle157Thr) | heterozygous |
| 300 | *CHEK2* | 3 | c.470T>C | p.(lle157Thr) | heterozygous |
| 301 | *CHEK2* | 3 | c.539G>A | p.(Arg180His) | heterozygous |
| 302 | *CHEK2* | 3 | c.1383C>G | p.(Asp461Glu) | heterozygous |
| 303 | *CHEK2* | 3 | c.740C>T | p.(Ala247Val) | heterozygous |
| 304 | *CHEK2* | 3 | c.1180G>A | p.(Glu394Lys) | heterozygous |
| 305 | *CHEK2* | 3 | c.470T>C | p.(lle157Thr) | heterozygous |
| 306 | *CHEK2* | 3 | c.1312G> T | p.(Asp438Tyr) | heterozygous |
| 307 | *CHEK2* | 3 | c.190G>A | p.(Glu64Lys) | heterozygous |
| 308 | *RAD51C* | 5 | c.1026+5_+7delGTA | not available | heterozygous |
| 309 | *RAD51C* | 5 | c.(837+1_838-1)_(904+1_905-1)del | p.(Glu303LysfsTer12) | heterozygous |
| 310 | *RAD51C* | 5 | c.224dup | p.(Tyr75Ter) | heterozygous |
| 311 | *RAD51C* | 5 | c.525dup | p.(Cys176LeufsTer27) | heterozygous |
| 312 | *RAD51C* | 5 | c.706-2A>G | not available | heterozygous |
| 313 | *RAD51C* | 4 | c.904+5G>T | not available | heterozygous |
| 314 | *RAD51C* | 4 | c.904+5G>T | not available | heterozygous |
| 315 | *RAD51C* | 3 | c.621T>A | p.(His207Gln) | heterozygous |
| 316 | *RAD51C* | 3 | c.335G>C | p.(Gly112Ala) | heterozygous |
| 317 | *RAD51C* | 3 | c.419T>A | p.(Val140Glu) | heterozygous |
| 318 | *RAD51C* | 3 | c.784T>G | p.(Leu262Val) | heterozygous |
| 319 | *RAD51C* | 3 | c.956G>A | p.(Arg319Gln) | heterozygous |
| 320 | *RAD51C* | 3 | c.548T>C | p.(lle183Thr) | heterozygous |
| 321 | *RAD51C* | 3 | c.431T>C | p.(lle144Thr) | heterozygous |
| 322 | *RAD51C* | 3 | c.454G>T | p.(Ala152Ser) | heterozygous |
| 323 | *RAD51C* | 2 | c.837T>A | p.(=) | heterozygous |
| 324 | *RAD51D* | 5 | c.357_360del | p.(Cys119TrpfsTer16) | heterozygous |
| 325 | *RAD51D* | 5 | c.357_360del | p.(Cys119TrpfsTer16) | heterozygous |
| 326 | *RAD51D* | 4 | c.1A>G | p.(Met1?) | heterozygous |
| 327 | *RAD51D* | 4 | g.(?_33427954)_33428227del | not available | heterozygous |
| 328 | *RAD51D* | 4 | c.577-1G>T | not available | heterozygous |
| 329 | *RAD51D* | 4 | g.(?_ 33427954)_(33428073_?)del | not available | heterozygous |
| 330 | *RAD51D* | 4 | c.898C>T | p.(Arg300Ter) | heterozygous |
| 331 | *RAD51D* | 4 | c.1A>G | p.(Met1?) | not available |
| 332 | *RAD51D* | 4 | c.1A>G | p.(Met1?) | heterozygous |
| 333 | *RAD51D* | 4 | c.1A>G | p.(Met1?) | heterozygous |
| 334 | *RAD51D* | 4 | c.1A>G | p.(Met1Val) | heterozygous |
| 335 | *RAD51D* | 3 | c.862G>A | p.(Gly288Ser) | heterozygous |
| 336 | *RAD51D* | 3 | c.223C>A | p.(Leu75Met) | heterozygous |
| 337 | *RAD51D* | 3 | c.791T>C | p.(Leu264Pro) | heterozygous |
| 338 | *RAD51D* | 3 | c.715C>G | p.(Arg239Gly) | heterozygous |
| 339 | *RAD51D* | 3 | c.715C>G | p.(Arg239Gly) | heterozygous |
| 340 | *RAD51D* | 3 | c.433C>T | p.(Arg145Cys) | heterozygous |
| 341 | *RAD51D* | 3 | c.823C>T | p.(Arg275Trp) | heterozygous |
| 342 | *RAD51D* | 3 | c.434G>A | p.(Arg145His) | heterozygous |
| 343 | *RAD51D* | 3 | c.34C>G | p.(Leu12Val) | heterozygous |
| 344 | *RAD51D* | 3 | c.796C>T | p.(Arg266Cys) | heterozygous |
| 345 | *RAD51D* | 3 | c.932T>C | p.(lle311 Thr) | heterozygous |

*Table S2*. Genetic alterations in the cohort.
